# The impact of increasing saline flush volume to reduce the amount of residual air in the delivery system of aortic prostheses—a randomized controlled trial

**DOI:** 10.3389/fcvm.2024.1335903

**Published:** 2024-03-22

**Authors:** András Szentiványi, Sarolta Borzsák, Milán Vecsey-Nagy, András Süvegh, Artúr Hüttl, Daniele Mariastefano Fontanini, Zoltán Szeberin, Csaba Csobay-Novák

**Affiliations:** ^1^ Department of Interventional Radiology, Semmelweis University, Budapest, Hungary; ^2^ Semmelweis Aortic Center, Heart and Vascular Center, Semmelweis University, Budapest, Hungary; ^3^ Department of Vascular and Endovascular Surgery, Semmelweis University, Budapest, Hungary

**Keywords:** EVAR, saline flushing, air embolism, stroke, TEVAR, perigraft air

## Abstract

**Background:**

Air embolism is a known risk during thoracic endovascular aortic repair (TEVAR) and is associated with an incomplete deairing of the delivery system despite the saline lavage recommended by the instructions for use (IFU). As the delivery systems are identical and residual air remains frequently in the abdominal aortic aneurysm sac, endovascular aortic repair (EVAR) can be used to examine the effectiveness of deairing maneuvers. We aimed to evaluate whether increasing the flush volume can result in a more complete deairing.

**Methods:**

Patients undergoing EVAR were randomly assigned according to flushing volume (Group A, 1× IFU; Group B, 4× IFU). The Terumo Aortic Anaconda and Treo and Cook Zenith Alpha Abdominal stent grafts were randomly implanted in equal distribution (10-10-10). The quantity of air trapped in the aneurysm sac was measured using a pre-discharge computed tomography angiography (CTA). Thirty patients were enrolled and equally distributed between the two groups, with no differences observed in any demographic or anatomical factors.

**Results:**

The presence of air was less frequent in Group A compared to that in Group B [7 (47%) vs. 13 (87%), *p* = .02], and the air volume was less in Group A compared to that in Group B (103.5 ± 210.4 vs. 175.5 ± 175.0 mm^3^, *p* = .04). Additionally, the volume of trapped air was higher with the Anaconda graft type (*p* = .025).

**Discussion:**

These findings suggest that increased flushing volume is associated with a higher amount of trapped air; thus, following the IFU might be associated with a reduced risk of air embolization. Furthermore, significant differences were identified between devices in terms of the amount of trapped air.

**Clinical trial registration:**

[NCT04909190], [ClinicalTrials.gov].

## 1 Introduction

Stroke is a significant complication of thoracic endovascular aortic repair (TEVAR), with reported rates ranging from 2% to 8% (1, 2). Besides debris embolization originating from the aortic wall due to catheterization and device manipulation across the arch, air embolization plays a major role in intraprocedural cerebral embolism during TEVAR (3, 4) and other endovascular interventions involving the aortic arch (5). The source of the air entering the bloodstream is the stent graft delivery system, where air bubbles become trapped between the folds of the stent graft, which is loaded into a large delivery catheter under dry conditions in ambient air at the factory and released when the graft is opened (6, 7). To reduce undesirable air embolization from endovascular devices, their delivery systems often include channels for saline flushing prior to insertion to displace air.

An incomplete deairing of the delivery system associated with standard volume flushing with normal saline is widely acknowledged. A number of different approaches were used in the hope of a more complete deairing and thus a reduction in the rate of air embolism, such as normal volume of saline supplemented by carbon dioxide (CO_2_) and perfluorocarbon lavage (6–10). In routine clinical practice, many clinicians have adopted the practice of flushing 120 ml of saline to reduce the volume of ambient air in the sheath, regardless of the type of graft being used. This amount of saline is approximately four times higher than the amount prescribed in the instructions for use (IFU), which is concordant with previous results (11, 12). To evaluate the effect of the different flushing techniques, abdominal endovascular aneurysm repair (EVAR) is often used instead of TEVAR (6, 9, 13).

This single-center, randomized study aimed to compare saline flushing with normal (1× IFU) and increased volume (4× IFU) during standard EVAR, focusing on the amount of air present in the aneurysm sac as measured on pre-discharge computed tomography (CT).

### 1.1 Study design

This was a randomized controlled trial conducted at a tertiary center involving patients who underwent EVAR. The study adhered to the CONSORT reporting guidelines (14) and is registered with ClinicalTrials.gov (NCT04909190 on 1 June 2021). Ethical approval was obtained from the Semmelweis University Regional and Institutional Committee of Science and Research Ethics 94/2021, and the study was conducted in accordance with the Declaration of Helsinki. In accordance with the current guidelines on the management of aortic abdominal aneurysm (AAA), pre-discharge CTA is part of the institutional protocol; therefore, the study participants did not have any extra exposure to radiation (15). Informed consent was obtained to publish follow-up images and additional information in an online open-access publication.

### 1.2 Participants and setting

Consecutive EVAR candidates of a tertiary center who underwent standard EVAR for AAA disease between 11 June 2021 and 11 April 2022 were enrolled. The inclusion criteria were on label suitability for all three EVAR devices (including the main body graft with limb grafts of the same factory) with a flush port used at our institution: Cook Zenith Alpha Abdominal (Cook Medical, Bloomington, IN, USA) and Terumo Aortic Treo and Anaconda (Terumo Aortic, Inchinnan, UK), particularly regarding anatomical conditions, age >18 years, and informed consent. The exclusion criteria were compromised proximal or distal landing zone requiring either fenestrated EVAR; the use of endoanchors; an additional graft component besides the main body, ipsilateral limb, and contralateral limb; an iliac branch device; or surgeons' disagreement with all three graft types due to their former experiences. All procedures were performed by the same, experienced endovascular team (16–19) in the same hybrid operating room, equipped with GE Discovery IGS 730 (GE HealthCare, Chicago, IL, USA), a fixed-mount imaging system. The scrub nurse performed deairing of the contrast media injector as per the IFU. Lege artis aspiration and flushing were performed each time a contrast injection was performed through any catheter or sheath. Immediately prior to insertion, the nurse was asked to flush the delivery system with the requested amount of normal saline through the flush port with a 20 ml syringe. In terms of the Anaconda graft type, the lavage volume was 30 ml or 120 ml, whereas in the two other conditions, 20 ml or 80 ml were flushed. The three-way stopcock was closed and was only opened when the flushing syringes had been already connected and saline was being flushed. For every flushing procedure, the tip of the delivery system was elevated by 40°. While resting, the graft was lying horizontally on the operating table. The following steps of the implantation were carried out just as a standard EVAR procedure, so all the devices, i.e., catheters, balloons, and sheaths, and also the ipsi- and contralateral limbs were flushed with saline per the IFU.

### 1.3 Data sources

Pre-discharge CTA was performed in every case per the institutional protocol. CTA images were transformed into a dedicated software tool (IntelliSpace Portal Version 9.0.4, Philips Healthcare, The Netherlands) for the quantitative measurement of the volume of the trapped air within the aneurysm sac. First, volume rendering was performed. Then the software automatically highlighted and measured all the air in the volume using a Hounsfield unit-based algorithm. Volume clipping, using both the 3D and 2D images, was utilized to cut away unwanted parts of the volume (such as extracorporeal air, air in the lungs, and air in the gastrointestinal system). The remaining air volume, which was in the aneurysm sac, was registered. This semiautomated measurement was performed by one reader (SB), who was blinded to the type of stent graft and amount of flushing volume. For five randomly selected patients, the measurement was repeated, and test–retest repeatability was assessed using the intraclass correlation coefficient (ICC).

### 1.4 Randomization

**Figure 1:**
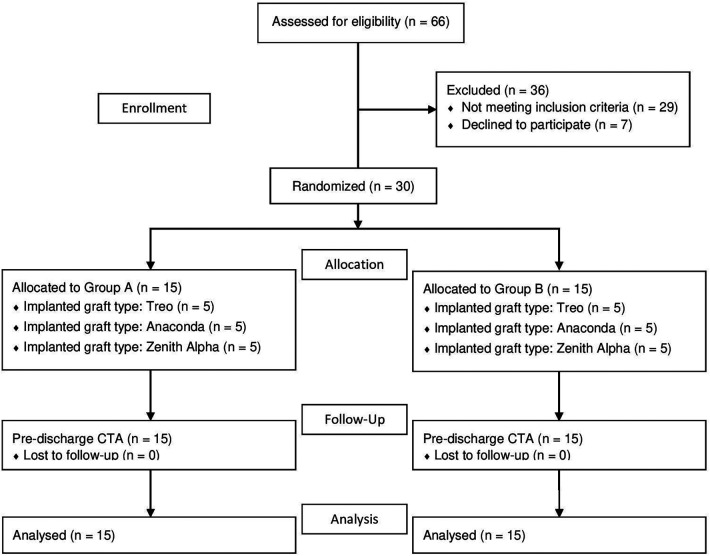
CONSORT flowchart of the study of saline flushing to reduce the amount of residual air inside a stent graft delivery system. The patients were randomized into two groups. Group A receiving saline volume equal to the instruction for use (IFU) and Group B receiving saline volume quadruple to that prescribed in the IFU.

An independent scientific assistant enrolled the patients in this prospective study and randomized them into two groups: Group A received saline volume equal to that prescribed in the IFU, and Group B received saline quadruple to that prescribed in the IFU. Randomization was performed on an equal basis in blocks of five using sealed envelopes stating group and type of graft (Group A-Treo, Group B-Treo, Group A-Anaconda, Group B-Anaconda, Group A-Zenith Alpha, Group B-Zenith Alpha). Out of the total 30 patients, half were assigned to Group A and the other half to Group B. Furthermore, within a single graft type, an equal number of patients were assigned to Groups A and B (Figure 1).

### 1.5 Statistical analysis

Data are presented as mean ± standard deviation for continuous variables and as numbers and percentages for categorical variables. The normality of continuous parameters was tested with the Shapiro–Wilk test. In the case of normal distribution, groups were compared using an unpaired *t*-test or Mann–Whitney *U*-test for data failing tests of normality. The categorical variables were compared by Pearson's chi-squared test. Kruskal–Wallis rank sum tests were used to analyze for differences between groups. Univariate linear regression analysis was used to evaluate the determinants of trapped air volume. To assess independent predictors, the variables with a *p* value of <.10 were entered into multivariate linear regression. The expected frequency of the presence of trapped air was 85% in Group A and 35% in Group B, based on our clinical evidence in the absence of previous studies. With a .05 significance level and 80% power, a total of 28 patients (14 in each group) were needed per treatment arm. To ensure adequate power for this trial, taking into account missing information or dropouts, 15 patients were included in each treatment arm. A two-sided *p* < .05 was considered to be significant. Data analysis was performed using SPSS Statistics 28 software (IBM, Armonk, NY, USA).

## 2 Results

### 2.1 Descriptive data

A total of 66 patients were prospectively enrolled in the trial between 10 June 2021 and 30 June 2022. Nine patients declined to participate, while an additional 27 patients were excluded for various reasons: the use of endoanchors (*n* = 3); additional graft component implantation besides the main body, ipsilateral limb, and the contralateral limb (*n* = 18); complex aortic intervention like fenestrated or branched EVAR (*n* = 5); or the surgeons' disagreement with all three graft types (*n* = 1). Thirty patients were randomized in the study, and all study participants underwent successful EVAR procedures, an uneventful postoperative period, and a pre-discharge CTA. Half of the patients received treatment with a quadruple-volume flushed graft with equal distribution among the three devices. There were no significant differences observed in age, sex ratio, demographical risk factors, premedication, medical history, or aneurysm characteristics between Groups A and B (Table 1). Table 1 summarizes the morphological and procedural data and the results of postoperative trapped gas analysis in both groups. All implantations were performed using three components: a main body, an ipsilateral limb, and a contralateral limb.

**Table 1:** Baseline demographics, anatomical factors, and characteristics for 30 patients with endovascular aneurysm repair (EVAR). Flush volume equal or quadruple to the recommended volume in the instruction for use (IFU) of the specific device. Data are presented as *n* (%) or mean ± standard deviation.

	Group A (1× IFU) *n* = 15	Group B (4× IFU) *n* = 15	*p* value*
Demographics			
Age—year	71.8 ± 7.4	70.6 ± 6.3	.49
Male gender	13 (87)	13 (87)	1.0
Body mass index—kg/m^2^	27.5 ± 5.9	26.2 ± 3.7	.069
Smoking	7 (47)	7 (47)	1.0
Hypertension	11 (73)	13 (87)	.36
Diabetes	3 (20)	1 (7)	.28
Hypercholesterolemia	4 (27)	5 (33)	.69
CKD 3–5	1 (7)	0 (0)	.31
History of stroke	2 (13)	0 (0)	.14
Malignancy	4 (27)	5 (33)	.69
COPD	2 (13)	3 (20)	.62
Heart failure	3 (20)	4 (27)	.67
Anatomical factors			
Mean aneurysm size (mm)	61.5 ± 13.4	60.6 ± 8.1	.66
Mean lumen size of aneurysm (mm)	43.8 ± 10.2	43.5 ± 15.3	.085
Medical treatment			
Antiplatelet	12 (80)	10 (67)	.41
Anticoagulant	3 (20)	2 (13)	.62
Statin	11 (73)	7 (47)	.20
Antihypertensive	10 (67)	12 (80)	.41
Procedural data			
**Volume of trapped air (mm^3^)**	**103.5 ± 210.4**	**175.5 ± 175.0**	**.** **04**
**Presence of trapped air**	**7** **(47)**	**13** **(87)**	**.** **02**
Air kerma (mGy)	711.5 ± 836.6	454.1 ± 415.6	.66
Length of stay (day)	3.7 ± 1.0	4.1 ± 4.0	.31
Days until CTA (day)	3.5 ± .74	4.8 ± 3.8	.40
Volume of trapped air for graft types			
Treo (mm^3^)	56.3 ± 92.1	62.6 ± 69.5	.74
Anaconda (mm^3^)	183.1 ± 338.6	328.8 ± 217.1	.25
Zenith Alpha (mm^3^)	71.1 ± 140.8	241.8 ± 135.2	.17

CKD, chronic kidney disease; COPD, chronic obstructive pulmonary disease; CTA, computed tomography angiography.

Bold indicates statistical significance.

*p values at .05 were considered statistically significant.

### 2.2 Outcome data

**Figure 2:**
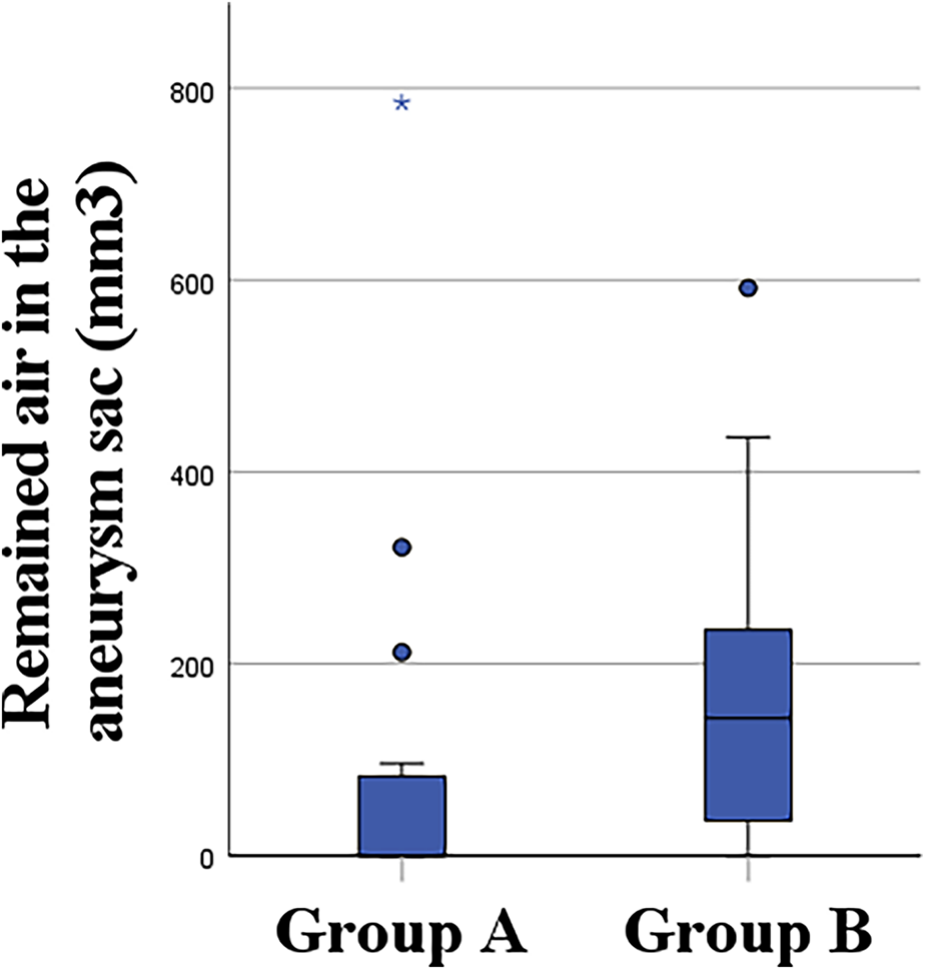
Quantitative measurement of the volume of remaining air in Group A and Group B (Group A, 1×IFU; Group B, 4× IFU).

Trapped air volume and the presence of trapped air were significantly higher (*p* = .04 and *p* = .02) in group B (quadruple flushing). The volume of trapped air was lower (103.5 ± 210.4 vs. 175.5 ± 175.0) in Group A using standard amounts of saline (Figure 2). Subgroup analysis based on device types showed no significant differences in air kerma (614.2 ± 895.3; 460.0 ± 315.6; 674.7 ± 689.8), trapped air volume (59.4 ± 77.0; 256.0 ± 279.0; 103.2 ± 115.7), and endoleak type II [in 6 (67%); 3 (30%); 7 (78%) patients] between the Terumo Aortic Treo, Terumo Aortic Anaconda, and Cook Zenith Alpha devices, accordingly. Further details are provided in Table 2. ICC demonstrated excellent repeatability of 1.0.

**Table 2:** Subgroup analysis based on device type.

	Terumo Treo *n* = 10	Terumo Anaconda *n* = 10	Cook Zenith Alpha *n* = 10	*p* value*
Age—year	68.9 ± 6.0	73.3 ± 7.9	71.4 ± 6.3	.40
Body mass index—kg/m^2^	27.3 ± 5.1	28.3 ± 4.0	25.0 ± 5.4	.39
Mean aneurysm size	62.1 ± 9.6	59.1 ± 3.8	62.0 ± 16.5	.69
Mean lumen size of aneurysm	49.3 ± 10.6	40.5 ± 14.6	41.1 ± 12.1	.17
Days until CTA	3.6 ± .70	3.2 ± 1.3	4.9 ± 4.8	.86
Length of stay	3.8 ± .63	4.0 ± 1.1	4.7 ± 4.7	.41
Air kerma (mGy)	614.2 ± 895.3	460.0 ± 315.6	674.7 ± 689.8	.84
Volume of trapped air	59.4 ± 77.0	256.0 ± 279.0	103.2 ± 115.7	.16
Presence of trapped air	5 (50)	8 (80)	7 (70)	.35
Endoleak type II	6 (67)	3 (30)	7 (78)	.086

CTA, computed tomography angiography.

*p values at. 05 were considered statistically significant.

### 2.3 Secondary outcome

No clinically significant complications due to air embolism were observed. Demographical, anatomical, interventional, procedural, and anamnestic factors were assessed with univariable regression analysis (Table 3), which showed Terumo Aortic Anaconda graft type as a significant variable (*p* = .017) on trapped air. This factor and hypertension were analyzed with multivariate linear regression, revealing Anaconda (*p* = .039) as an independent risk factor.

**Table 3:** Uni- and multivariate linear regression analysis of anatomical and interventional factors of the volume of trapped air.

	Univariate				Multivariate			
	*β*	95% CI, lower-upper		*p*	*β*	95% CI, lower-upper		*p*
Anatomical factors								
Aneurysm diameter	1.84	−5.0	8.7	.59				
Flow lumen diameter	1.13	−4.7	7.0	.67				
Patent inferior mesenteric artery	−26.2	−192.6	140.1	.75				
Endoleak	−59.9	−215.6	95.9	.44				
Stent graft type								
Treo	−120.2	−269.4	29.1	.11				
**Anaconda**	**174**.**7**	**33**.**8**	**315**.**6**	.**017**	**154.8**	**21.1**	**288**.**5**	.**025**
Zenith Alpha	−54.5	−209.4	100.4	.48				

Bold indicates statistical significance (*p* < .05).

## 3 Discussion

Stroke is a common complication of TEVAR. Although theoretically, device manipulation over the aortic arch could easily result in embolization from the dislodged aortic wall thrombus and/or debris, it is believed that a significant percentage of the emboli are indeed gaseous—air bubbles trapped in the delivery system and released during deployment. This idea was confirmed by Bismuth et al. in 2011 when they found that transcranial Doppler during zone 0–2 TEVAR detected the highest rate of embolic event during device deployment and not during wire manipulation (3).

To investigate the parameters associated with the rate of air embolism, endovascular aortic repair (EVAR) is increasingly utilized as a clinical model for several reasons: (1) the delivery systems of EVAR devices are almost identical to that of TEVAR implants; (2) air bubbles at the outer surface of the prosthesis can be trapped within the abdominal aortic aneurysm sac at the luminal surface of the ventral mural thrombus, which is a usual and easily quantifiable finding on the first follow-up CT (20, 21); and (3) EVAR is more frequently performed than TEVAR (9, 13). Similar to TEVAR, hepatic failure, renal failure, or adverse cardiovascular events can occur due to perigraft air after EVAR, and trapped air also should be monitored to rule out the possibility of graft infection (20, 21). Trapped air detected in the aneurysm sac after EVAR has an identical etiology to what may result in stroke during a TEVAR procedure, thus making EVAR an appropriate model to evaluate the effectiveness of delivery system deairing.

In the last few years, numerous attempts have been made to reduce residual air. Kölbel et al. (8) introduced the CO_2_ flushing technique before saline lavage, adhering to the volume prescribed in the IFU. In 2016, this study demonstrated the safety of the method, and the results suggested its effectiveness. However, it missed the comparison with the control group. Rohlffs et al. (6) demonstrated, for the first time *in vitro*, that extra CO2 flushing may lead to significantly reduced trapped air. Their images clearly show that ambient air remains between the folds of the thoracic stent graft. Three years later in 2019, Rohlffs et al. (7) published another *in vitro* study about remaining air reduction in a water-filled container. A significant decrease of trapped air was achieved with perfluorocarbon flush after the prescribed volume of saline flushing. The result was observed mostly with big bubbles at the tip of the sheath. One year earlier, Saleptsis et al. (13) were the first to describe the correlation between larger perfused lumen diameter and the presence of remaining air after EVAR. Types of graft were also studied, and EVAR was assessed with a higher risk of the presence of air than fenestrated EVAR or branched EVAR. This finding is in accordance with the research of Eleshra et al. (9), reporting a retrospective analysis of differences in the presence of gas in the aneurysm sac after EVAR. Introducing extra CO2 flushing into the protocol led to a lower incidence of trapped air in EVARs and also a decreased volume of air on the control CTA scans. Anatomical predictors, such as perfused lumen diameter and aneurysm size, were associated with the presence of remaining gas. In 2019, Rylski et al. (11) determined in an *in vitro* study that 120 ml saline flush instead of 40 ml saline flush leads to a significantly lower remaining air in the delivery system of a TEVAR. Two years later, a 120 ml flush of 0.9% heparinized saline solution seemed to minimalize neurological events after TEVAR in a retrospective study by Branzan et al. (12). Our study investigates air embolism in EVAR, and our findings should be translated into practice in the use of TEVAR with caution.

This randomized controlled trial is a pioneer study comparing different volumes of saline flush. Our results show that, contrary to what one could expect, an increased saline flush volume may not be associated with a decrease in the volume of trapped air inside the delivery system of an aortic prosthesis. This gives further evidence that the use of these devices in accordance with the IFU might be associated with a lower complication rate.

Although the syringe was connected with a three-way stopcock to avoid entering extra air, the increased air intake during the operation was likely attributable to the multiple syringe changes. Rinse was presumably always performed at a higher pressure in the delivery system than in the operating room, so water and air could only come outwards. Contrary to an injector, manual flushing did not provide constant pressure.

A significant difference in the volume of trapped air was also found between the three devices used. Terumo Aortic Anaconda graft type was found to be independently associated with a higher volume of trapped air. This is most likely linked to a higher volume of lavage due to the higher recommended volume in the IFU and also to differences between the flush port design of the devices. In an experimental setting, Rohlffs et al. provided evidence that an additional flush port may significantly reduce the amount of trapped air. Further investigations regarding the optimal flush port design may lead to a more complete deairing. On the other hand, the components and structure of stent grafts differ: the Anaconda has a special metal core made up of a single metal wire that takes the shape of a spring when opened. This design suggests that perhaps a larger volume of air can be squeezed in.

Without being statistically significant, hypertension has a trend toward being associated with more remaining air (Table 3).

If we look at Groups A and B of each graft type separately, Treo stands out in terms of volume of trapped air, with practically no difference between the two groups (56.3 ml in Group A and 62.6 ml in Group B). In comparison, the Anaconda and Zenith Alpha graft types show a remarkable but not significant difference. It should be mentioned that these two stent grafts differ from Treo in the sterilization protocol, as Anaconda and Zenith Alpha are sterilized with ethylene oxide (EtO), and Treo is gamma-radiated. But after EtO sterilization, the remaining EtO–gas ratio is less than 250 ppm (22). During gamma radiation, no extra gas component is used. Consequently after both of these procedures, the composition of the trapped air is practically identical to ambient air. Thus, the theoretical difference in the water solubility of the trapped gas is unlikely to be relevant regarding the amount of air detected on CT. However, this has not yet been investigated, and our study suggests that it may be worth evaluating in a future publication.

Several studies (4, 6–8) have raised similar questions regarding the reduction of air using a flushing technique, with some employing CO_2_. Based on the IFU, saline lavage is obligatory. Consequently in all studies, a solution was applied to the flushing chamber after extra CO2 lavage. These studies employed two techniques to minimize the amount of intravascular air bubbles. Therefore, a comparison between ours and theirs may not be appropriate. Eleshra et al. (9) compared gas and saline flushing techniques and observed a lower ratio of trapped air in the case of extra gas flush. This finding was associated with better penetration of CO2, due to constant pressure during flushing, and the flushing did not require reconnections and disconnections to the flushing port.

### 3.1 Limitations

We acknowledge several limitations in this study. The number of performed EVARs was relatively small. We only included polyethylene terephthalate (PETE)-based prostheses, although it is unlikely that the effect of saline flush volume increase is different in other devices with expanded polytetrafluoroethylene (ePTFE) coating. Although we tried to make the flushing protocol as strict as possible to reduce the risk of human error, there is still room for improvement regarding saline flushing. Flushing with the use of a power injector at a fixed pressure, eliminating the need to change a syringe during flushing could further increase reproducibility. However, questions remain regarding the applicability of implementing this approach in everyday practice. The temperature of saline and length of time between flushing and implantation may also be a confounding factor, neither being controlled nor evaluated in this current study.

There may be a slight bias in our results stemming from the investigation of graft types prepared with more than one type of sterilization technique. Since the flushing process simply involves a hub-to-tip filling of a large sheath with saline injected from the distal end, outside of the device, and there is no available data to support whether flushing is not equally effective on the outer and the inner side of the fabric, we assumed that trapped air in the aneurysm sac (most likely originating from the outer side of the graft after incomplete flushing) can be used to monitor the effectivity of the deairing process, to reduce the stroke rate of TEVAR.

## 4 Conclusion

Air embolism during EVAR continues to pose challenges despite ongoing efforts to minimize the volume of trapped air. This study represents the first randomized controlled trial comparing different flush volumes. Following the IFU instead of increasing saline volume may lead to a lower risk of air embolization. A significant difference was also found between the endografts used, suggesting that flush port design could serve as an additional tool to achieve a more efficient deairing of delivery systems.

## Data Availability

The raw data supporting the conclusions of this article will be made available by the authors, without undue reservation.
